# Flux periodic oscillations and phase-coherent transport in GeTe nanowire-based devices

**DOI:** 10.1038/s41467-021-21042-5

**Published:** 2021-02-02

**Authors:** Jinzhong Zhang, Pok-Lam Tse, Abdur-Rehman Jalil, Jonas Kölzer, Daniel Rosenbach, Martina Luysberg, Gregory Panaitov, Hans Lüth, Zhigao Hu, Detlev Grützmacher, Jia Grace Lu, Thomas Schäpers

**Affiliations:** 1 Peter Grünberg Institut (PGI-9), Forschungszentrum Jülich, Jülich, 52425 Germany; 2 JARA-Fundamentals of Future Information Technology, Jülich-Aachen Research Alliance, Forschungszentrum Jülich and RWTH Aachen University, Jülich, 52425 Germany; 3 Technical Center for Multifunctional Magneto-Optical Spectroscopy (Shanghai), Engineering Research Center of Nanophotonics & Advanced Instrument (Ministry of Education), Department of Materials, School of Physics and Electronics Science, East China Normal University, Shanghai, 200241 China; 4 Department of Physics and Astronomy and Department of Electrophysics, University of Southern California, Los Angeles, 90089 CA USA; 5 Ernst Ruska Center, Forschungszentrum Jülich, Jülich, 52425 Germany; 6 Institute of Complex Systems (ICS-8) Forschungszentrum Jülich, Jülich, 52425 Germany

**Keywords:** Electronic devices, Nanoscale materials

## Abstract

Despite the fact that GeTe is known to be a very interesting material for applications in thermoelectrics and for phase-change memories, the knowledge on its low-temperature transport properties is only limited. We report on phase-coherent phenomena in the magnetotransport of GeTe nanowires. From universal conductance fluctuations measured on GeTe nanowires with Au contacts, a phase-coherence length of about 280 nm at 0.5 K is determined. The distinct phase-coherence is confirmed by the observation of Aharonov–Bohm type oscillations for parallel magnetic fields. We interpret the occurrence of these magnetic flux-periodic oscillations by the formation of a tubular hole accumulation layer. For Nb/GeTe-nanowire/Nb Josephson junctions we obtained a critical current of 0.2 *μ*A at 0.4 K. By applying a perpendicular magnetic field the critical current decreases monotonously with increasing field, whereas in a parallel field the critical current oscillates with a period of the magnetic flux quantum confirming the presence of a tubular hole channel.

## Abstract (web summary)

A deep understanding of low-temperature transport properties of GeTe material remains a challenge. Here, the authors investigate phase-coherent phenomena in GeTe nanowire structures where the occurrence of magnetic flux-periodic oscillations come from the formation of a tubular hole accumulation layer.

## Introduction

In the past decades, GeTe-based alloys attracted an increasing interest for their applications in optical data storage and phase change memories due to a reversible rapid transformation between amorphous and crystalline phase^1,2^, and a large contrast of optical constants and electrical conductivity for the two phases^3^. In addition, GeTe-rich alloys can be used in intermediate temperature thermoelectric applications for converting waste heat to electrical energy because of their high thermoelectric performance, high thermal and mechanical stability^4,5^. From the fundamental physics perspective it is important to unravel its intrinsic property governed by the unique metavalent bonding and to shed light on the microscopic mechanisms that contribute to its phase change property^6^.

Pristine GeTe is a *p*-type semiconductor with a trigonal ferroelectric phase (space group *R*3*m*, No. 160) and an indirect band gap of *E*_g_ ~ 0.61 eV^4,7–9^. Only recently it was recognized that its trigonal ferroelectric phase makes this material very attractive for spinelectronic applications^8^. This is due to the fact that the non-centrosymmetric structure with displaced adjacent Ge and Te layers along the [111] direction exhibit a net dielectric polarization. The inversion symmetry breaking as a result of the displacement leads to a giant Rashba spin splitting of the bulk bands in GeTe^8,10–12^. In fact, photoemission spectroscopy measurements confirmed the Rashba band splitting as well as the corresponding spin polarization^13–16^. As a further verification of the presence of Rashba spin–orbit interaction, weak antilocalization was observed in GeTe layers in low-temperature magnetotransport experiments. In addition to spin-related properties GeTe is observed to be superconducting at temperatures below 0.3 K^17–19^. Recently, Narayan et al.^20^ have investigated the non-equilibrium superconductivity in GeTe Hall bar samples with a semiconductor-superconductor transition temperature of about 0.14 K and a critical magnetic field of around 70 mT. Last but not least, the combination of strong spin–orbit coupling and superconductivity makes GeTe a very attractive candidate for topological systems^19,21^.

To explore possible device applications the transport properties of quasi one-dimensional GeTe nanowires have been investigated. Here, it turned out that GeTe nanowires are very interesting as building blocks in phase-change memories^22–24^. However, to find out about the suitability of GeTe for the above mentioned spintronic and quantum device applications low-temperature quantum transport measurements on nanostructures are required. In order to do so, we have grown GeTe nanowires and measured the transport properties of GeTe nanowires contacted with normal metals as well as GeTe nanowire-based Josephson junctions. For the normal contacted nanowires we investigated phase-coherent transport phenomena such as universal conductance fluctuations, weak antilocalization, as well as Aharonov–Bohm-type resistance oscillations. For the GeTe nanowires equipped with Nb contacts the proximity-induced superconductivity as well as critical temperature and magnetic field of Nb/GeTe-nanowire/Nb Josephson junctions are studied.

## Results and discussion

**Fig. 1:**
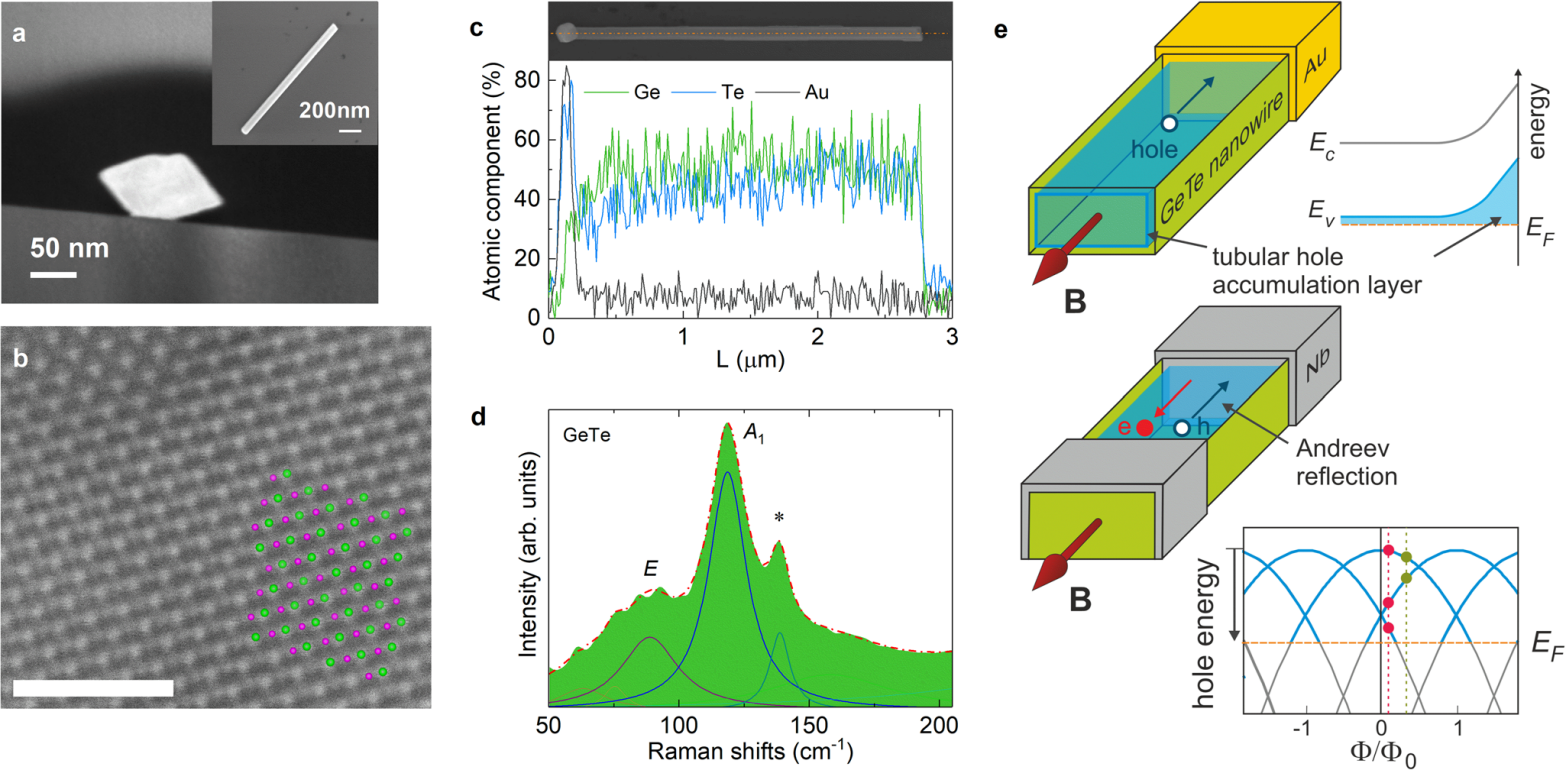
Structural characterization and schematics of carrier transport. **a** Scanning transmission electron micrograph of GeTe NW in cross section. **b** HAADF image of GeTe NW cross section obtained via aberration corrected STEM with evident trigonal crystal structure, where light green atoms represent Te and magenta colored atoms indicate the position of Ge atoms. Scale bar: 2 nm. **c** A SEM image of a GeTe nanowire and the corresponding atomic composition along the nanowire from Energy dispersive X-ray spectroscopy. **d** Experimental and fitted Raman spectra (solid lines) with mode assignments of a GeTe single nanowire. *E* and *A*_1_ are the first-order Raman-active modes while the feature (*) arises from surface oxidation. **e** Top: Schematics of a GeTe nanowire with a tubular hole accumulation layer and a magnetic field along the wire axis. The scheme on the right shows the upwards bending of the bands at the surface, with *E*_F_ the Fermi energy as well as *E*_c_ and *E*_v_ the conduction band bottom and valence band top, respectively. Bottom: schematics of a GeTe nanowire contacted with Nb electrodes. At the interface Andreev reflection occurs. The schematics on the right shows the energy spectrum of hole states in a tubular conductor for different angular momenta as a function of the normalized flux Φ/Φ_0_ for *E*_kin_ = 0. Note that for hole states the energy increases downwards and the effective mass is positive. The red and green dots indicate the occupied channels at different flux values.

GeTe nanowires (NWs) were synthesized by a Au-catalyzed vapor-liquid-solid growth in a tube furnace with GeTe powders^22,25^. The as-grown GeTe NWs on a Si/SiO_2_ substrate are straight with a length of more than 5 μm. The cross section for the GeTe nanowires has a rhombic shape with a side length of about 80 nm (cf. Fig. 1a). A high-angle annular dark field (HAADF) image of GeTe NW cross section was obtained via aberration corrected scanning transmission electron microscopy (STEM). In the corresponding image shown in Fig. 1b one can identify a trigonal crystal structure. The positions of the Te and Ge atoms are indicated by light green and magenta dots, respectively. For device fabrication the GeTe NWs were transferred to a Si/SiO_2_ substrate. Subsequently, superconducting (Nb) or normal-metal (Ti/Au) contact electrodes were prepared by using electron beam lithography and magnetic sputtering.

In order to investigate the material properties of the nanowires energy dispersive X-ray spectroscopy (EDX) and Raman spectroscopy were performed. The EDX results shown in Fig. 1c reveal that the as-grown GeTe has an ideal atomic ratio of Ge:Te = 1:1 and the element component are uniform along the NWs except the nanowire ends with a Au nanoparticle, typical of vapor-liquid-solid growth. Furthermore, Raman spectra of a GeTe single nanowire demonstrate unambiguously that GeTe NWs exhibit rhombohedral phase (*R3m*) at room temperature (cf. Fig. 1d). According to the group theory, the two peaks near 89 and 119 cm^−1^ are assigned to the first-order Raman-active modes *E*(TO) and *A*_1_(LO), respectively^26,27^. Theoretically, *E* and *A*_1_ relates to the bending modes of the GeTe_4_ tetrahedra and symmetric stretching vibration of the Te–Te bond, respectively^28^. Note that the peak labeled by the symbol (*) at around 140 cm^−1^ arises from surface oxidation^29^. Other peaks/shoulders may originate from the breaking of inversion symmetry due to vacancies, defects, and distortion of bonds^30^.

**Fig. 2:**
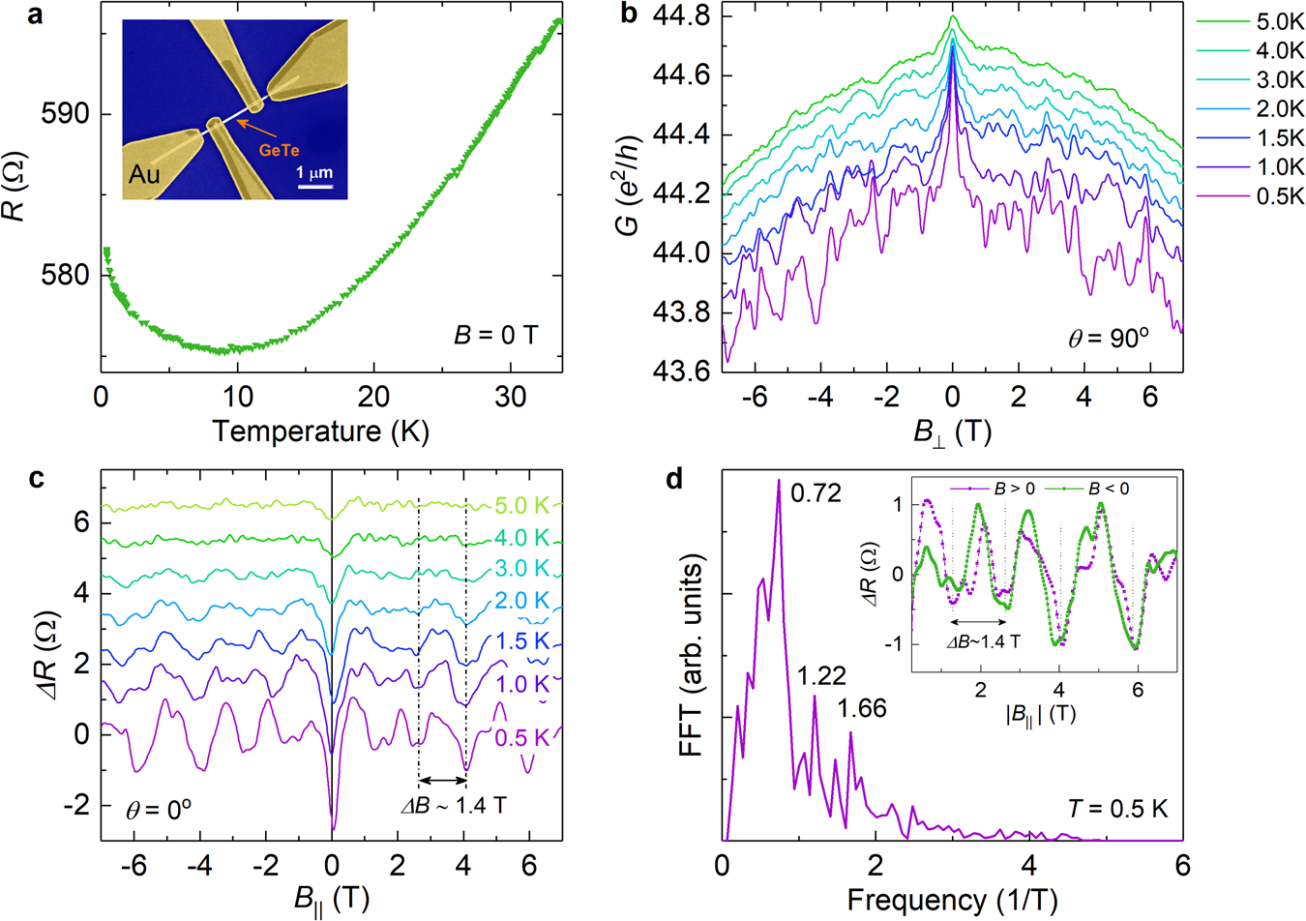
Magnetotransport of a normal contacted GeTe nanowire. **a** Four-terminal resistance as a function of temperature at zero magnetic field of a Au/GeTe-nanowire/Au device. Inset: Scanning electron microscopy image of a GeTe NW contacted with Ti/Au leads. **b** Conductance in units of *e*^2^/*h* as a function of *B*_⊥_ at temperatures from 0.5 to 5.0 K. **c** Magnetoresistance oscillations after subtracting a parabolic background as a function of *B*_∣∣_ at temperatures from 0.5 to 5.0 K. **d** Fast Fourier transform (FFT) of the magnetoresistance oscillations in a parallel magnetic field at 0.5 K. The inset shows the corresponding resistance oscillations for the positive and negative magnetic field range.

In order to gain information on the electrical properties of the GeTe nanowires we measured the magnetotransport of samples equipped with normal contacts (Ti/Au: 20 nm/100 nm). An electron beam micrograph of the contacted 80-nm-wide nanowire is shown in Fig. 2a (inset). The measurements were performed in a four-terminal configuration with the bias current supplied at the outer contacts. The width and separation of the inner electrodes is about 0.5 and 1.0 μm, respectively. In Fig. 2a the resistance as a function of temperature is plotted. One finds that the resistance decreases with decreasing temperature in the temperature range from 35 to 10 K, and then increases monotonously upon further decrease of the temperature down to 0.4 K. The temperature dependence corresponds to a metallic behavior, where the upturn in resistance for temperatures below 10 K can be attributed to the electron–electron interaction and weak localization effects^31–33^. No transition to a superconducting behavior is observed, probably because the lowest temperature of 0.4 K is larger than the critical temperature^17,19^.

Figure 2b shows the conductance *G* in units of *e*^2^/*h* with the magnetic field applied perpendicular to the nanowire axis (*B*_⊥_, *θ* = 90°), at temperatures between 0.5 and 5.0 K. Here, *e* is the electron charge and *h* is the Planck constant. Similarly to GeTe films^19^, a pronounced peak is observed at zero magnetic field which is attributed to the weak antilocalization effect. This feature originates from interference effects of holes in the presence of spin–orbit coupling. Indeed, for GeTe a bulk Rasbha effect in the valence band is theoretically predicted^8,13^. Furthermore, as discussed in more detail below, band bending at the surface in connection with according potential gradients may also result in spin–orbit coupling effects. The conductance traces shown in Fig. 2b reveal a modulation pattern, which can be assigned to universal conductance fluctuations^34^. Their amplitude decreases with increasing the temperature mainly due to the reduction of the phase-coherence length^35^. From the autocorrelation function of the magnetoconductance, given by *F*(Δ*B*) = 〈*G*(*B*)*G*(*B* + Δ*B*)〉 − 〈*G*(*B*)〉^2^, information on the phase-coherence length *l*_ϕ_ can be obtained^34^. Here, 〈…〉 is the average over the magnetic field. For quasi one-dimensional systems, i.e., systems with *l*_ϕ_ larger than the width of the wire, the correlation field *B*_c_ defined by *F*(*B*_c_) = *F*(0)/2, is inversely proportional to the phase-coherence length *l*_ϕ_: ${B}_{{\rm{c}}} \sim \sqrt{3}{{{\Phi }}}_{0}/(\pi {l}_{\phi }d)$, with the magnetic flux quantum given by Φ_0_ = *h*/*e* (see also Supplementary Fig. 5 in Supplementary Note 2)^36,37^. For the fluctuations measured at 0.5 K, we extracted a correlation field of *B*_c_ = 0.10 T. Taking the nanowire width of *d* ~ 80 nm into account we obtain a phase-coherence length of about 280 nm. The presence of conductance fluctuations and the fact that *l*_ϕ_ is considerably larger than the width of the nanowire confirms that the transport takes place in the diffusive quasi one-dimensional regime.

Figure 2c shows the resistance Δ*R* after subtraction of a slowly varying background as a function of a magnetic field *B*_∣∣_ applied along the nanowire axis. For this magnetic field orientation one finds a regular oscillation pattern with an oscillation period of about Δ*B* = 1.4 T (cf. Fig. 2d, inset). This is also confirmed by the fast Fourier spectrum of the measurement at 0.5 K depicted in Fig. 2d. Here, a pronounced peak is found at a frequency of 0.72 T^−1^, corresponding to the aforementioned period of 1.4 T. In addition, some features at 1.22 and 1.66 T^−1^ are present.

We attribute the regular oscillations to Aharonov–Bohm type oscillations^38^ originating from transport in tubular hole states in a conductive surface accumulation layer penetrated by a magnetic flux Φ (cf. Fig. 1e, upper left schematics)^35,39–42^. The involvement of phase-coherent tubular states is supported by the large phase-coherence length being comparable to the circumference of the nanowire. We assume that in case of our GeTe nanowire, the holes accumulated in the surface channel are supplied from surface states. In fact, such a surface accumulation layer is mainly observed for narrow band gap materials, e.g., for InAs. Similar but inverted as in the case of InAs the GeTe electronic band structure exhibits some lower valence band maxima^43,44^, which may be responsible for the charge neutrality level of the surface states to be located within the bulk valence band. As a consequence, a tubular hole accumulation layer is formed at the surface^45^. Owing to the hole accumulation the valence band is bent upwards at the interface (cf. Fig. 1e and Supplementary Note 3 for more details on the formation of the accumulation layer).

In order to describe the transport through the tubular hole channel with a rectangular cross section in more detail, we assume a circular cross section^42,46^. In both cases quantized states will be formed, where the wave function along the circumference has to match after a full cycle. However, in case of a circular cross section the state components perpendicular to the nanowire axis can be described by coherent angular momentum states, which can be ordered according to their angular momentum quantum number *l* of their angular momentum *L*_z_. This simplifies the theoretical description considerably. In the presence of a magnetic flux through the wire cross section the energy eigenstates are given by^39,41^

1 $$E={E}_{{\rm{kin}}}+\frac{{\hslash }^{2}}{2{m}^{* }{r}_{0}^{2}}{\left(l-\frac{{{\Phi }}}{{{{\Phi }}}_{0}}\right)}^{2}\ ,$$

with *E*_kin_ the kinetic energy along the wire, m^*^ the effective hole mass, and *r*_0_ the radius of the tubular channel. The energy is periodic in Φ_0_ = *h*/*e*.

A schematics of the energy spectrum is given in Fig. 1e, bottom. In a ballistic picture the conductance is determined by the number of occupied hole channels above the Fermi level *E*_F_, e.g., three channels for a small flux (red dots in the schematics) and two channels for a slightly larger flux (green dots)^39^. Thus, when the magnetic field is increased, the number of occupied hole channels changes periodically with the period of Φ_0_. In the non-ballistic diffusive case, a similar flux-periodic modulation is expected as well^47^. However, it must be stressed that the actual presence of the Aharonov–Bohm oscillations does not depend on the hole concentration in the surface gas.

From the measured period of Δ*B* = 1.4 T of our nanowire we can deduce the expected area *S* encircled by the surface channel from Φ_0_ = *S* × Δ*B*, which results in a value of about *S* = 3000 nm^2^, corresponding to a diameter of approximately 60 nm for a circular cross section. For our nanowire with a width of 80 nm, we estimate an area of about 5000 nm^2^ assuming a circular cross section. Obviously, the expected cross section is smaller than the actual geometrical cross sectional area of our GeTe nanowire. The discrepancy may be attributed to the fact that the nanowire surface is oxidized so that the hole accumulation layer is slightly pushed inside. Furthermore, the radial component wave function usually has an extension of a few nanometers, which also leads to a reduction of the effective cross sectional area. As mentioned above, some weaker maxima at higher frequencies, i.e., at 1.22 and 1.66 T^−1^, are found in the Fourier spectrum. These features may be related to higher harmonics or to Altshuler–Aronov–Spivak oscillations comprising a *h*/2*e* periodicity^48^.

**Fig. 3:**
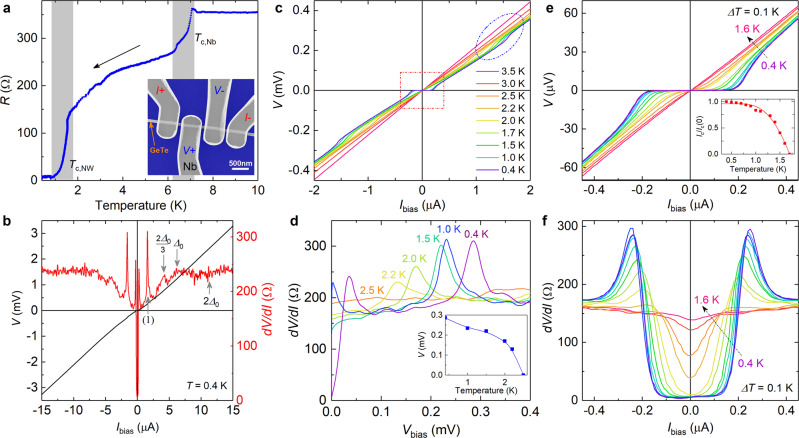
Transport characteristic of Nb/GeTe-nanowire/Nb junction at zero magnetic field. **a** Resistance *R* of a Nb/GeTe-nanowire/Nb junction during a cooling process. There are two transitions (*T*_c,Nb_ and *T*_c,NW_) labeled by vertical shadows. Inset: A scanning electron microscopy image of a Nb/GeTe-nanowire/Nb junction. **b** *I*–*V* characteristics and corresponding d*V*/d*I* trace of a GeTe-based junction in a high bias current range at 0.4 K. The subgap features at 2Δ_0_/3 and Δ_0_ as well as the location of 2Δ_0_ are indicated by arrows. Furthermore, an additional sharp feature is found at (1). **c** *I*–*V* curves as a function of large bias current range. The superconducting range at small bias currents is indicated by an rectangle and the step feature at around ±1.5 μA can be found within the ellipse. **d** Corresponding d*V*/d*I* traces as a function of bias voltage at various temperatures. Inset: Position as a function of temperature of the peak assigned feature (1) indicated in **b**. **e** *I*–*V* characteristics and **f** the corresponding d*V*/d*I* curves in a small bias current range at temperatures between 0.4 and 1.6 K. The inset of **e** is the experimental (dots) and fit (solid line) *I*_c_/*I*_c_(0) as a function of temperature.

We now turn to measurements of GeTe nanowires equipped with superconducting contacts. The inset of Fig. 3a shows a GeTe NW-based junction device with Nb contacts. The nanowire has a width of 80 nm, while the width and separation of Nb leads are about 500 and 90 nm, respectively. The outer Nb leads are employed to apply bias current, while the inner pair of contacts serve as voltage probes^49,50^. Figure 3a shows the resistance *R* at zero magnetic field as a function of temperature. The measurement shown in Fig. 3a indicates that the Nb electrodes have a transition temperature of about *T*_c,Nb_ = 7.0 K. From the relation of the *T*_c,Nb_ ≈ 3.9, with *k*_B_ the Boltzmann constant and critical temperature *T*_c,Nb_, we estimate the Nb energy gap Δ_0_ to be about 1.2 meV at zero temperature^51,52^. Moreover, there is another drop in the temperature region between 1.0 and 2.0 K, which is assigned to the superconducting transition temperature *T*_c,NW_ of the Nb/GeTe-NW/Nb junction. The two-step feature has been observed before in Josephson junctions with a normal metal^53^ and a semiconductor weak link^54^ as well as in topological insulator-based junctions^55^. The normal state four-terminal resistance (*R*_N_) of the present Nb/GeTe-NW/Nb junction is about 355 Ω at temperatures above *T*_c,Nb_.

Figure 3b shows the current–voltage (*I*–*V*) characteristics and corresponding differential resistance (d*V*/d*I*) curve measured at 0.4 K. At low bias currents a superconducting state is observed with a critical current of 200 nA. In the d*V*/d*I* trace modulations are observed, which we assign to subharmonic gap structure caused by multiple Andreev reflections, as described within the Octavio–Tinkham–Blonder–Klapwijk (OTBK) model^56,57^. As indicated in Fig. 3b, peaks corresponding to 2Δ_0_/3 and Δ_0_ are present, while in the region around 2Δ_0_ one only finds a broad indentation. Although relatively weak, the observed subharmonic gap features basically follow the expected modulations predicted by the OTBK model. Several reasons can be made out to be responsible for the dampening of the subharmonic gap structures. First of all, the finite junction length in relation to the superconducting coherence length can cause a weakening of the subharmonic gap structures^58^. Furthermore, in our structure the interface between the Nb and GeTe is laterally not sharply defined, i.e., the effective junction length might vary. This probably also leads to a broadening of the subharmonic gap structures. In addition to the regular subharmonic gap structure we also found sharp peak indicated by (1) in Fig. 3b. We attribute this feature to a parasitic superconducting switching event, probably due to some defect in the superconducting electrodes in series.

As a further step, the temperature dependence of the *I*–*V* characteristics is investigated (cf. Fig. 3c). Obviously, the superconducting range at small bias currents (indicated by a rectangle) shrinks and finally vanishes upon increasing the temperature from 0.4 to 3.5 K. The same is true for the step feature at around ±1.5 μA. Regarding the latter, the corresponding *dV*/d*I* traces as a function of bias voltage are shown in Fig. 3d for temperatures in the range from 0.4 to 2.5 K. One finds that the feature corresponding to peak (1) in Fig. 3b shifts towards smaller bias voltages and eventually vanishes at a temperature of 2.5 K (cf. Fig. 3d, inset). The strong temperature dependence confirms that this feature is directly related to the superconducting state.

Figure 3e shows the *I*–*V* characteristics of the GeTe-NW based Josephson junction in a small bias current range at temperatures ranging from 0.4 to 1.6 K. At 0.4 K the critical current *I*_c,NW_ is about 200 nA. As the temperature increases, the superconducting plateau diminishes and disappears at about 1.6 K. The according corresponding differential resistance traces are shown in Fig. 3f. The dependence of normalized critical current *I*_c_/*I*_c_(0) as a function of temperature are depicted in the inset of Fig. 3e. The temperature dependence of the supercurrent can be assigned to a metallic diffusive junction^59^. However, since no reliable data on the diffusion constant and Thouless energy can be obtained, we have refrained from a detailed analysis.

**Fig. 4:**
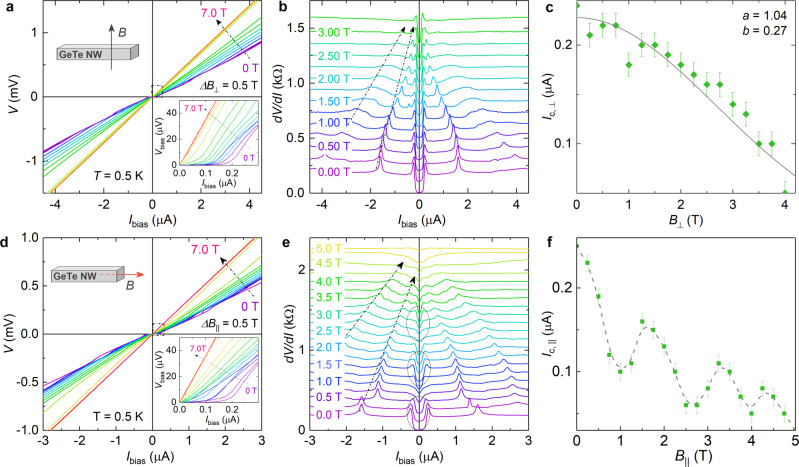
Magnetotransport transport in Nb/GeTe-nanowire/Nb junction. **a** *I*–*V* characteristics and **b** the corresponding d*V*/d*I* traces of a Nb/GeTe-NW/Nb junction as a function of bias current at 0.5 K by applying various perpendicular magnetic fields *B*_⊥_. The insets in **a** show the orientation of the magnetic field and a detail at small bias currents. **c** Experimental (dots) and fitted (solid line) critical current I_*c*,⊥_ as a function of magnetic field (*B*_⊥_) based on the d*V*/d*I* curves at a small bias current region (cf. inset in **a**). The error bars represent the resolution of the current bias. **d** *I*–*V* characteristics and **e** the corresponding d*V*/d*I* traces as a function of bias current at 0.5 K by applying various magnetic field parallel to the GeTe NW axis (*B*_∣∣_). The insets in **d** show a details of the characteristics around zero current bias and illustrate the magnetic field orientation with respect to the nanowire. All d*V*/d*I* curves in **b** and **e** are shifted vertically for clarity. In **e** the ranges where a supercurrent is observed are indicated by the ellipses. **f** Critical current *I*_c,∣∣_ as a function of magnetic field based on the d*V*/d*I*. The dashed line is a guide to the eye.

The Nb/GeTe–NW/Nb junctions were measured in a magnetic field in two orientations, i.e., parallel to the nanowire axis and perpendicular to the substrate plane. For the perpendicular configuration the critical field *B*_c,⊥_ was determined to be around 4 T while for the parallel case we got *B*_c,∣∣_ ≈ 5 T. (In Supplementary Note 4 measurements at various tilt angles of the magnetic field are shown.) Figure 4a, b depict the evolution of *I*–*V* and corresponding d*V*/d*I* characteristics of the Nb/GeTe–NW/Nb junction in a perpendicular magnetic field (*B*_⊥_) at 0.5 K. With increasing magnetic field, the supercurrent is suppressed and then disappears, as can be seen in more detail in Fig. 4a (inset). The features at around ±1.6 and ±4 μA found in the d*V*/d*I* trace at zero field shift towards zero bias upon increasing the magnetic field. The shift can be attributed to the reduced superconducting gap when a magnetic field is applied. As shown in Fig. 4c, we find a monotonous decrease of the critical current *I*_c,⊥_ with increasing *B*_⊥_. A complete suppression of *I*_c,⊥_ occurs at around 4 T. A similar monotonous decrease of critical current with *B*_⊥_ was observed in planar Nb/Au/Nb^53^ as well as in semiconductor nanowire based Josephson junctions^52,60^. It can be explained within the framework of a theoretical model for the proximity effect in diffusive narrow-width Josephson junctions^61^. Within that model the decay of *I*_c,⊥_ can be described by^53,61^: ${I}_{{\rm{c}},\perp }(B)={I}_{{\rm{c}},\perp }(0)\cdot a{e}^{-b{(BS/{{{\Phi }}}_{0})}^{2}}$. Here, *I*_*c*,⊥_(0) is the critical current at *B* = 0 T. The *a* and *b* are fit parameters and *S* is the effective area (80 nm × 90 nm) of the Nb/GeTe/Nb junction perpendicular to magnetic field. In the model we do not distinguish between bulk and surface contributions. The fit curve from theory (solid line) agrees well with the experimental data for fit parameters *a* and *b* of 1.04 and 0.27, respectively.

In the case that the applied magnetic field is parallel to the GeTe wire axis (*B*_∣∣_), the supercurrent is modulated when the magnetic field is increased, as can be seen in Fig. 4d, e. Furthermore, similar to the previous case where *B*_⊥_ was applied the peak features in the d*V*/d*I* traces shift towards zero bias upon increasing of *B*_∣∣_. In Fig. 4e the ranges where a Josephson supercurrent is present is indicated. The corresponding values of *I*_c,∣∣_ as a function of *B*_∣∣_ are plotted in Fig. 4f. One clearly finds an oscillatory behavior of *I*_c,∣∣_. The oscillation period of Δ*B*_∣∣_ is very similar to the period of the sample with normal contacts, as discussed before. Since the cross section of the nanowire is very similar to the previous one, we can deduce that oscillations of *I*_c,∣∣_ are periodic with a single magnetic flux quantum Φ_0_ = *h*/*e*. We interpret the behavior by a Josephson supercurrent which is carried by coherent closed-loop states in the surface accumulation layer at the GeTe nanowire surface. As illustrated in Fig. 1e, bottom, the supercurrent originates from phase-coherent Andreev retro-reflections of tubular hole and electron states at both GeTe/Nb interfaces^62^. In each cycle a Cooper pair is effectively transported from one Nb electrode to the other one. In the measurements of GeTe nanowire with normal contacts, we found that the resistance was modulated with a magnetic flux quantum because of the periodic change of the number of occupied transport channels. In case of superconducting electrodes, the magnitude of the Josephson supercurrent is also considered to be determined by the number of hole and electron transport channels, hence a flux periodic modulation is expected as well. As a matter of fact, a magnetic flux modulation was found before in junctions based on GaAs/InAs core/shell nanowires^41,63^. However, in that case no clear supercurrent was observed and a period of Φ_0_/2 was found.

In summary, magnetotransport of GeTe nanowire-based devices have been investigated in a four-terminal configuration. The temperature dependent resistance of the normal metal contacted Au/GeTe-nanowire/Au devices reveals that GeTe exhibits a semiconducting behavior until 0.4 K. At low temperatures universal conductance fluctuations were observed which allowed us to extract the phase-coherence length *l*_*ϕ*_ being as large as 280 nm at 0.5 K. The weak antilocalization feature observed around zero magnetic field indicates the presence of spin–orbit coupling in the valence band. Even more, when a parallel magnetic field is applied regular Aharonov–Bohm type oscillations are found, which are attributed to the formation of a hole accumulation layer at the nanowire surface. In the case of Nb/GeTe–NW/Nb Josephson junctions, we observed a critical supercurrent of about 200 nA at 0.4 K. The temperature dependence of *T*_c,NW_ can be explained in the framework of a diffusive junction. With increasing a perpendicular magnetic field the critical current decreases monotonously. This feature can be explained within theoretical models covering the small junction limit. For magnetic fields applied parallel to the nanowire axis regular oscillations of the critical current are observed, which are attributed to a supercurrent carried by the surface accumulation layer in the GeTe nanowires. For future studies it would be interesting to find out if the transport phenomena based on a surface accumulation channel can also be observed in GeTe nanostructures with different geometries, e.g., zigzag nanowires or nanohelices^22,25^.

Our investigations showed that distinct phase-coherent phenomena can be observed in GeTe nanowire structures. The very pronounced and sharp weak antilocalization feature indicates that the corresponding spin precession length is very short, which implies that spin manipulation might be possible on a very small length scale^8^. So far, we could not discriminate between Rashba spin–orbit coupling originating from the potential gradient connected to the surface hole accumulation layer and the electric field due to the polarization in the bulk. In order to gain more insight in the contributions of the surface and the giant bulk Rashba effect, it would be very interesting to perform measurements on gated nanowires. This is also of interest with respect to the switching effect based on the ferroelectric properties of GeTe. All in all, the presence of spin–orbit coupling in combination with the superconducting proximity effect make these nanowires very attractive for applications in the field of topological quantum computation. In this context, it is also worth-mentioning that by combining GeTe with three-dimensional topological insulators in a superlattice the topological properties can be tailored^21^.

## Methods

### GeTe nanowire synthesis

The synthesis of GeTe nanowires are achieved by a Au-catalyzed vapor-liquid-solid growth. Firstly, the Si(100) substrates with a native oxide were cleaned with acetone, iso-propyl alcohol, and deionized water in an ultrasonic cleaning bath, and then treated in a piranha solution to remove organic residues. After that, the substrates were immersed in Au nanoparticles solution for a few min and rinsed with deionized water. Finally, bulk GeTe (99.99%, Sigma-Aldrich) was evaporated at the center of a horizontal tube furnace, and the reaction product was collected downstream on a Si/SiO_2_ substrate covered with colloidal Au nanoparticles. Specifically, the furnace was evacuated and purged three times with Ar gas. Then it was heated to 400 ^∘^C and persisted for 8 h. During this program, the Ar flow rate and the pressure in the quartz tube were about 140 sccm and 10 Torr, respectively.

### GeTe-based device fabrication

In order to contact a single GeTe nanowire with Nb or Au electrodes, the as-grown GeTe NWs were transferred to a Si/SiO_2_/Si_3_N_4_ substrate with predefined markers for electron beam lithography. A two-layer (copolymer/950 K) polymethyl methacrylate (PMMA) resist system was adopted to realized an ideal shape of Nb or Au leads. In order to obtain a transparent interface between electrodes and GeTe, the samples were exposed to an oxygen plasma to remove resist residues on the contact area. Before the deposition of the electrodes on a single nanowire in a magnetron sputter chamber with a DC power of 250 W, the samples were lightly cleaned by 45 s Ar^+^ plasma milling to remove native oxidation layers on the GeTe surface.

### Morphology and composition characterizations

The morphology and chemical composition of the GeTe nanowires was determined by SEM and EDX, respectively. In addition, a cross sectional specimen has been prepared by focused ion beam techniques. High-angle annular dark field imaging in the STEM has been employed to investigate the cross sectional shape of the nanowires. In addition, the crystalline structure has been investigated by x-ray diffraction and Raman scattering. (In Supplementary Note 1 additional information on the sample characterization is provided.)

### Magnetotransport measurements

The temperature and magnetic field dependent *I* − *V* characteristics and d*V*/d*I* curves were measured using a standard lock-in technology in a ^3^He cryostat with a base temperature of 0.4 K and a magnetic field range from −8 to 8 T. A four-terminal current driven geometry is employed to directly measuring the voltage drop across the inner section of GeTe nanowire between the nearest internal electrodes. The external pair of electrodes was used for current bias^55^. The differential resistance (d*V*/d*I*) is gained by superimposing a small AC current of 10 nA at 9.4 Hz on a DC bias current. Note that the applied bias current should be as small as possible to avoid electron heating and damage of the GeTe-based nanodevices.

## Supplementary information

Supplementary Information

## Data Availability

The data that support the findings of this study is available from the authors on a reasonable request. The source data underlying Figs. 1–4 and Supplementary Figs. 3, 6, and 8 are provided as a Source Data file from the Jülich DATA repository (10.26165/JUELICH-DATA/M1IQVG).

## Source data

Source Data
