# Supplementary material for: Flux periodic oscillations and phase-coherent transport in GeTe nanowire-based devices
Source: Nat Commun. 2021 Feb 2;12:754. doi: 10.1038/s41467-021-21042-5 (PMC7854721; doi:10.1038/s41467-021-21042-5)
Supplement: Supplementary file 1 — Supplementary Information [file 41467_2021_21042_MOESM1_ESM.pdf]

Supplementary Information: Flux periodic oscillations and phase-coherent transport in GeTe nanowire-based devices

Jinzhong Zhang,[1,2,3] Pok-Lam Tse,[4] Abdur-Rehman Jalil,[1,2] Jonas Kölzer,[1,2] Daniel Rosenbach,[1,2] Martina Luysberg,[5] Gregory Panaitov,[6] Hans Lüth,[1,2] Zhigao Hu,[3] Detlev Grützmacher,[1,2] Jia Grace Lu,[4] and Thomas Schäpers[1,2]

[1]*Peter Grünberg Institut (PGI-9), Forschungszentrum Jülich, 52425 Jülich, Germany*

[2]*JARA-Fundamentals of Future Information Technology, Jülich-Aachen Research Alliance, Forschungszentrum Jülich and RWTH Aachen University, Germany*

[3]*Technical Center for Multifunctional Magneto-Optical Spectroscopy (Shanghai), Engineering Research Center of Nanophotonics & Advanced Instrument (Ministry of Education), Department of Materials, School of Physics and Electronics Science, East China Normal University, Shanghai 200241, China.*

[4]*Department of Physics and Astronomy and Department of Electrophysics, University of Southern California, CA 90089, Los Angeles, USA*

[5]*Ernst Ruska Center, Forschungszentrum Jülich, 52425 Jülich, Germany*

[6]*Institute of Complex Systems (ICS-8) Forschungszentrum Jülich, 52425 Jülich, Germany*

(Dated: January 4, 2021)

SUPPLEMENTARY NOTE 1: SAMPLE CHARACTERIZATION AND FABRICATION

In Supplementary Fig. 1 a scanning electron micrograph of the as-grown nanowires is shown. In Supplementary Fig. 2 a high angle annular dark field (HAADF) image of a GeTe nanowire cross section is depicted.

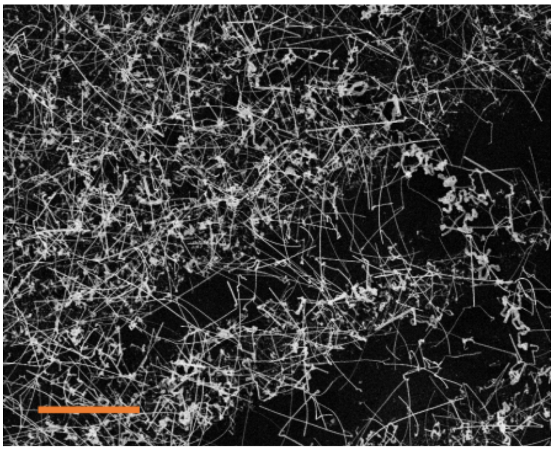

Supplementary Fig. 1: Scanning electron microscopy (SEM) image of the as-grown GeTe nanowires on a Si/SiO_2 substrate. The scale bar corresponds to 5 μm.

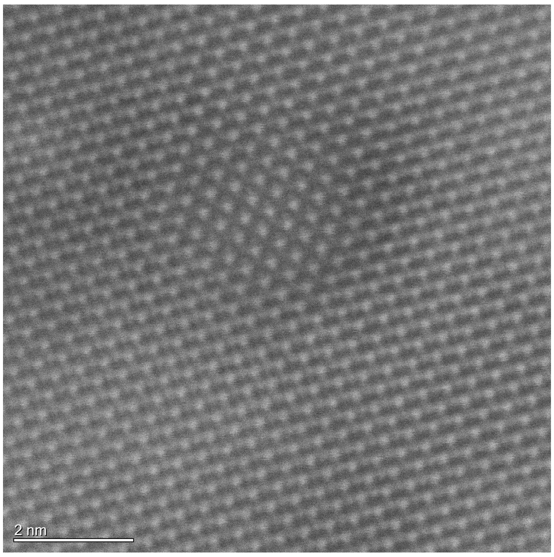

Supplementary Fig. 2: High angle annular dark field (HAADF) image of a GeTe nanowire cross section obtained via aberration corrected scanning transmission electron microscopy (STEM) with an evident trigonal crystal structure.

In Supplementary Fig. 3 an energy dispersive X-ray spectrum of a GeTe nanowire is shown. In the corresponding scanning electron micrograph (SEM) (inset) the spot where the spectrum is taken is indicated. The extracted atomic % values of Ge and Te are given in Supplementary Table I.

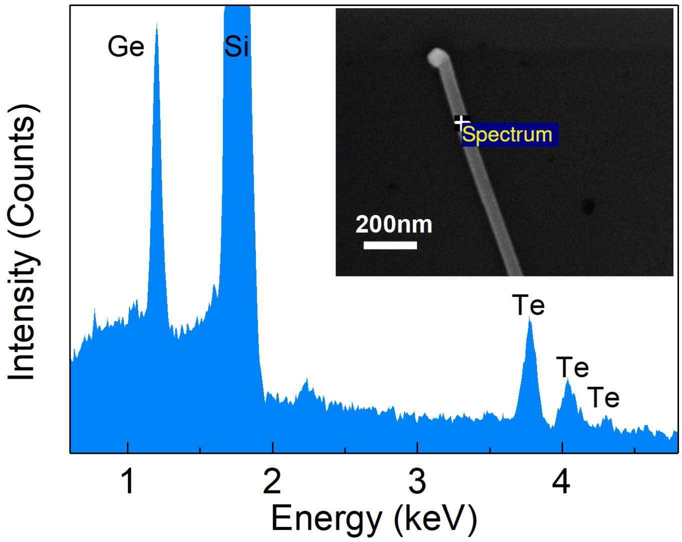

Supplementary Fig. 3: Energy dispersive X-ray spectrum of a GeTe nanowire and the corresponding SEM image.

element	atomic %
Ge L	49.12
Te L	50.88

Supplementary Table I: Atomic % obtained from the EDX spectrum.

Supplementary Fig. 4 shows a schematics of the 4-terminal measurement configuration. The current is supplied from the outer contacts, while the voltage drop is measured at the pair of inner contacts.

SUPPLEMENTARY NOTE 2: MAGNETOTRANSPORT MEASUREMENTS ON GOLD-CONTACTED NANOWIRES

In order to determine the correlation field B_c from the measurements of the conductance fluctuations the autocorrelation function $F(\Delta B) = \langle G(B)G(B+\Delta B)\rangle - \langle G(B)\rangle^2$ was

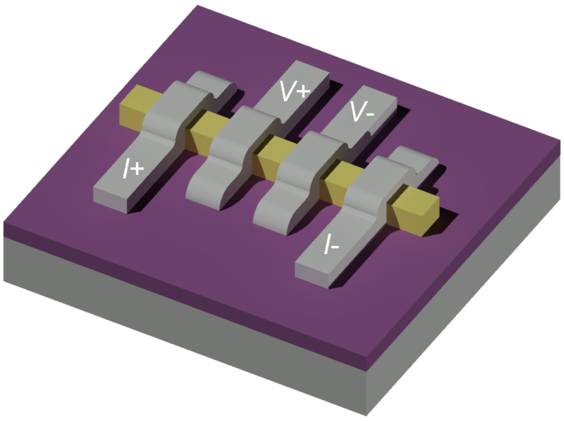

Supplementary Fig. 4: Schematics of the 4-terminal measurement configuration, with $I+$, $I-$ the current contacts and $V+$, $V-$ the voltage probes.

determined. In Supplementary Fig. 5 the normalized autocorrelation function is plotted for the magneto-conductance trace of the normal contacted GeTe nanowire at 0.5 K. The corresponding section of the conductance trace in the magnetic field interval from 0.5 to 7 T is shown in the inset of Supplementary Fig. 5. In order to exclude the contribution of the weak antilocalization feature the conductance close to zero magnetic field was left out. The correlation field B_c is extracted from the autocorrelation function with the condition $F(\Delta B)/F(0) = 0.5$. We find a value of 0.1 T, which corresponds to a phase-coherence length of about $l_\phi = 280\,\text{nm}$.

In addition to the measurements of the Au contacted GeTe nanowires in a parallel ($\theta = 0°$) and a perpendicular (90°) magnetic field, we also performed measurements where the magnetic field tilt angle was increased in steps from 0° to 90° (cf. Supplementary Fig. 6). One finds that the fluctuation pattern changes when the magnetic field is tilted. This can be explained by the fact that for the phase-coherent loops contributing to the fluctuation pattern the enclosed flux changes individually, depending on the spatial orientation of each loop. This results in a shift of the effective Aharonov–Bohm oscillation frequency of each loop. However, since each contributing loop has a different spatial orientation the frequency shift is different, thus resulting in a change of the overall fluctuation pattern [1, 2]. Within smaller ranges of tilt angles one can occasionally identify systematic shifts of some features (cf. dashed lines in Supplementary Fig. 6). In addition, the weak antilocalization feature around zero magnetic field also changes when the magnetic field orientation is varied. One finds that the resistance dip becomes narrower when the magnetic field is rotated towards

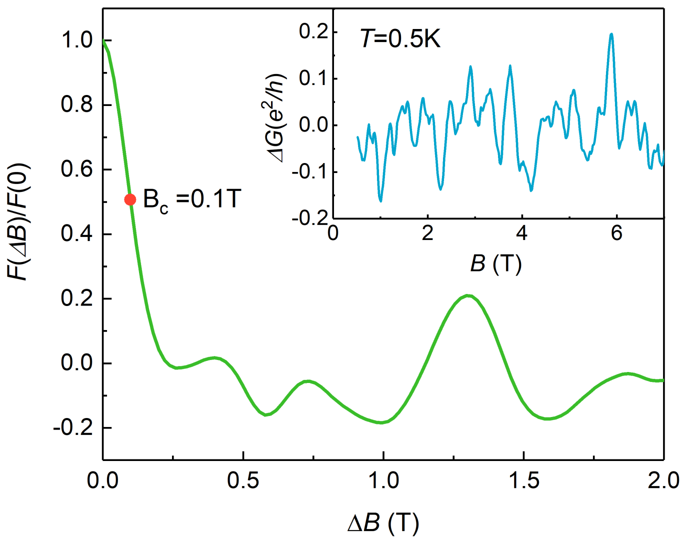

Supplementary Fig. 5: Normalized autocorrelation function $F(\Delta B)/F(0)$ as a function of ΔB of the Au/GeTe/Au device at 0.5 K. The magnetic field was oriented perpendicularly to the nanowire axis. The inset shows the according conductance window taken for the calculation of the autocorrelation function. The slowly varying background was subtracted.

the perpendicular direction. This behaviour can be explained by the according change of the characteristic magnetic dephasing length being responsible for the width of the weak antilocalization feature [2, 3].

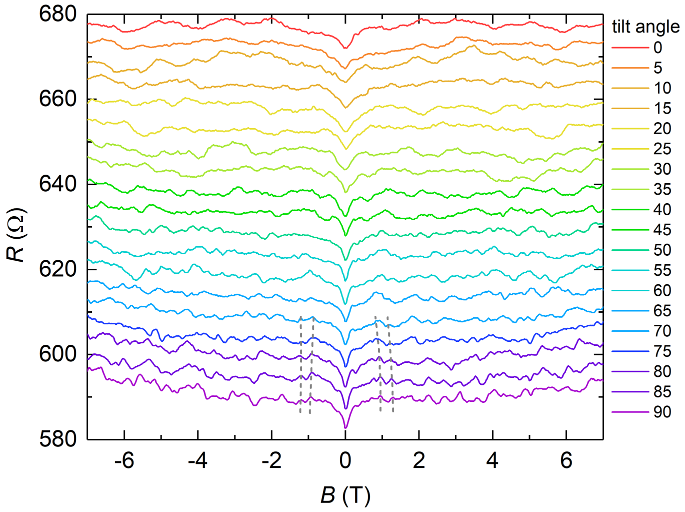

Supplementary Fig. 6: Resistance as a function of magnetic field amplitude of an Au/GeTe-nanowire/Au sample at various tilt angles θ of the magnetic field from 0° to 90°. The measurements were taken at a temperature of 0.5 K. The dashed lines trace some features in the resistance when the tilt angle is changed.

SUPPLEMENTARY NOTE 3: FORMATION OF SURFACE HOLE ACCUMULATION LAYER

The experimental results, i.e. a well-developed, clearly defined Aharonov–Bohm oscillation in the original ΔR data and in the Fourier spectrum, indicate a coherent spatially well-defined two-dimensional tubular conductance channel. The numerical estimation in the manuscript localizes this channel near the surface of the nanowire. Since no topological surface states are expected in the material, there is no other choice rather than assuming the existence of a two-dimensional carrier gas in a surface accumulation layer confined by the bending of the electronic bands. This band bending requires carrier exchange between surface states and the accumulation layer in the sub-surface bulk [4]. Surface states are derived from bulk electronic states and their density is usually high in spectral regions of flat bulk bands. In the case of the p-type GeTe material flat bulk valence band regions occur below the upper valence band edge [5, 6]. Donor type surface states derived from the bulk valence band are therefore expected below the upper valence band edge, while acceptor-type surface states derived from bulk conduction band states are expected in the spectral region of the bulk conduction band and below (cf. Supplementary Fig. 7). The surface state charge

neutrality level E_N, where acceptor-type surface states switch over into donor-type states is therefore expected considerably below the upper valence band edge. Since the Fermi level must be located close to the surface charge neutrality level E_N, an upwards band bending of the bulk valence band occurs with an accumulation of holes beneath the surface. The missing valence electrons are transferred into the acceptor-type surface states. There, a negative surface charge is build up, which compensates the postive charge of the holes in the accumulation layer [4].

These arguments are straightforwardly derived from the experimental results and from knowledge of the bulk electronic band structure [5, 6]. Details of the spectral distribution of the surface states and their density are unknown as in many other cases of common classical semiconductors. The density of surface states near the charge neutrality level, which directly determines the density in the two-dimensional hole accumulation layer is not known. Knowledge of these data requires extensive experimental surface studies which are far beyond the scope of the present manuscript. However, the essential details of the present analysis, in particular the Aharonov–Bohm frequency and the magnetic correlation field B_c, do not depend on the hole density in the accumulation layer and are therefore not affected by the lack of knowledge on the two-dimensional carrier density.

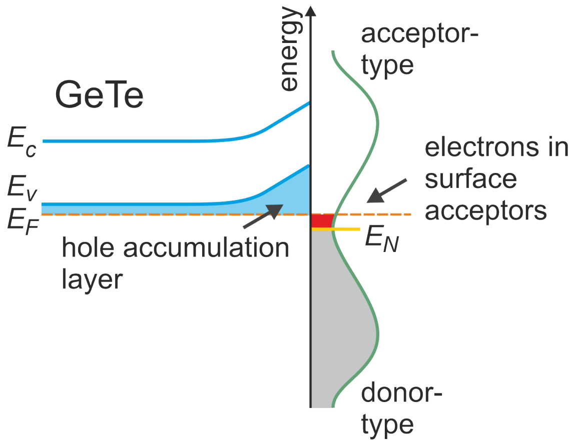

Supplementary Fig. 7: Schematics of the formation of a hole gas. Owing to the location of the neutrality level E_N below the valence band edge, electrons are transferred to the acceptor-type surface states creating a surface hole accumulation layer. Here, E_c and E_v are the conduction and valence band edges, respectively, while E_F is the Fermi energy.

SUPPLEMENTARY NOTE 4: MAGNETO-TRANSPORT MEASUREMENTS ON NIOBIUM-CONTACTED NANOWIRES

The differential resistance dV/dI of a Nb/GeTe-nanowire/Nb junction was measured at different tilt angles of the magnetic field (cf. Supplementary Fig. 8a). On finds a two-step transition to the normal state, which we attribute to the switching of the junction and of the Nb electrodes respectively. The transitions are shifted to larger magnetic fields when tilting towards the inplane magnetic fields. The corresponding temperature dependencies for $\theta = 90°$ and $0°$ are depicted in Supplementary Figs. 8b and c, respectively. From these measurements the corresponding critical magnetic fields for the Nb electrodes are extracted an plotted in Supplementary Fig. 8d.

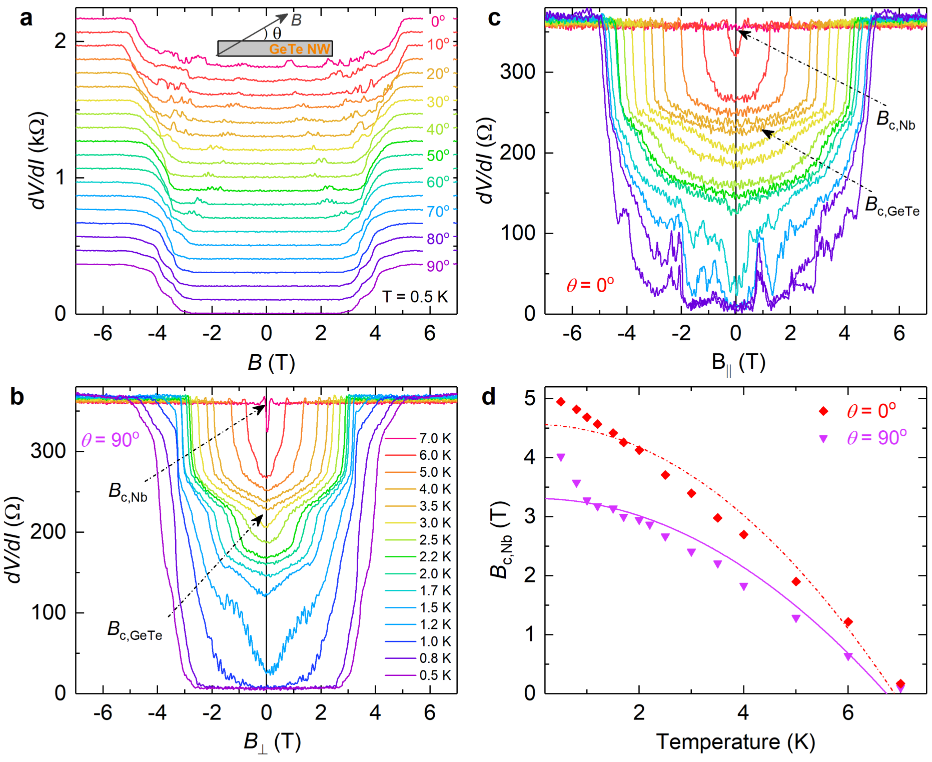

Supplementary Fig. 8: **a** dV/dI of a Nb/GeTe-nanowire/Nb junction for different tilt angles θ between magnetic field direction and nanowire axis at the temperature of 0.5 K. The inset depicts the definition of θ and the curves are shifted vertically for clarity. Temperature dependent dV/dI as a function of magnetic field at the angle θ of **b** 90° and **c** 0° at temperatures from 0.5 to 7.0 K. **d** Temperature dependence of critical magnetic field $B_{c,Nb}$ of Nb contacts in the applied magnetic field at the angles θ of 0° and 90°.

SUPPLEMENTARY REFERENCES

[1] Jespersen, T. S., Hauptmann, J. R., Sørensen, C. B. & Nygård, J. Probing the spatial electron distribution in InAs nanowires by anisotropic magnetoconductance fluctuations. *Phys. Rev. B* **91**, 041302 (2015).

[2] Kölzer, J. *et al.* Phase-coherent loops in selectively-grown topological insulator nanoribbons. *Nanotechnology* **31**, 325001 (2020). URL https://doi.org/10.1088%2F1361-6528%2Fab898a.

[3] Liang, D., Du, J. & Gao, X. P. A. Anisotropic magnetoconductance of a InAs nanowire: Angle-dependent suppression of one-dimensional weak localization. *Phys. Rev. B* **81**, 153304 (2010). URL https://link.aps.org/doi/10.1103/PhysRevB.81.153304.

[4] Lüth, H. *Surfaces, Interfaces and Thin Films* (Springer-Verlag, Berlin, 2015), 6th edn. URL http://www.springer.com/de/book/9783319107554#aboutBook.

[5] Polatoglou, H. M., Theodorou, G. & Economou, N. A. Band structure of cubic and rhombohedral GeTe. In Gornik, E., Heinrich, H. & Palmetshofer, L. (eds.) *Physics of Narrow Gap Semiconductors*, 221–225 (Springer Berlin Heidelberg, Berlin, Heidelberg, 1982).

[6] Jain, A. *et al.* The materials project: A materials genome approach to accelerating materials innovation. *APL Mater.* **1**, 011002 (2013). URL https://materialsproject.org/materials/mp-938/.
